# OA31 Successes and challenges in harmonising 4 national Juvenile Idiopathic Arthritis cohorts: an example from CLUSTER consortium

**DOI:** 10.1093/rap/rkac066.031

**Published:** 2022-09-28

**Authors:** Saskia Lawson-Tovey, Samantha Smith, Nophar Geifman, Stephanie Shoop-Worrall, Sandra Ng, Michael Barnes, Lucy Wedderburn, Kimme Hyrich

**Affiliations:** Centre for Genetics and Genomics Versus Arthritis, The University of Manchester, Manchester, United Kingdom; National Institute of Health Research Manchester Biomedical Research Centre, Manchester Academic Health Science Centre, Manchester University NHS Foundation Trust, Manchester, United Kingdom; Centre for Genetics and Genomics Versus Arthritis, The University of Manchester, Manchester, United Kingdom; School of Health Sciences, Faculty of Health and Medical Sciences, The University of Surrey, Guildford, United Kingdom; Centre for Epidemiology Versus Arthritis, The University of Manchester, Manchester, United Kingdom; Centre for Health Informatics, The University of Manchester, Manchester, United Kingdom; Centre for Translational Bioinformatics, William Harvey Research Institute, Bart's and the London School of Medicine and Dentistry, Queen Mary University London, London, United Kingdom; Centre for Translational Bioinformatics, William Harvey Research Institute, Bart's and the London School of Medicine and Dentistry, Queen Mary University London, London, United Kingdom; Centre for Adolescent Rheumatology Versus Arthritis at UCL, University College London Hospitals and Great Ormond Street Hospital, London, United Kingdom; Infection, Inflammation, and Rheumatology, UCL Great Ormond Street Institute of Child Health, London, United Kingdom; NIHR Great Ormond Street Hospital Biomedical Research Centre, London, United Kingdom; Centre for Epidemiology Versus Arthritis, The University of Manchester, Manchester, United Kingdom; National Institute of Health Research Manchester Biomedical Research Centre, Manchester Academic Health Science Centre, Manchester University NHS Foundation Trust, Manchester, United Kingdom

## Abstract

**Introduction/Background:**

The CLUSTER consortium aims to identify biomarkers and strata that improve personalised treatments for JIA/JIA-uveitis. By bringing together knowledge and data, CLUSTER can conduct novel analyses in this rare, heterogeneous disease. Data harmonisation across existing JIA cohorts facilitates new, larger datasets that would otherwise take years to collect; however, challenges exist as datasets are often collected autonomously. Here we present progress towards a large-scale, unique JIA data resource, bringing together treatment data from 4 real-world JIA treatment studies.

**Description/Method:**

Four studies (CAPS, CHARMS, BCRD and BSPAR-ETN; the latter two being part of the UK JIA Biologics register) contributed data into CLUSTER.

We created two clinical datasets of JIA patients starting first-line methotrexate (MTX) or tumour necrosis factor inhibitors (TNFi). Variables were selected based on a previously developed core dataset, accounting for different levels of granularity across studies. The same inclusion and exclusion criteria were agreed for both datasets, designed to allow for combined analysis of these.

OpenPseudonymiser software encrypted NHS numbers - these were matched cross-study to identify duplicates and checked against known duplicate lists. Errors in NHS numbers and existing duplicate matches were identified and corrected. Each NHS number was assigned a CLUSTER ID, meaning 1 child has the same ID across all relevant studies such that children contributing similar data across multiple studies could be identified.

**Discussion/Results:**

A total of 7013 records (from 5435 individuals) were identified; of which 2882 (41%, corresponding to 1304 individuals) represented the same child across >1 study. 197 individuals had duplicate records within 1 study, 961 in 2 studies, 142 in 3, and 4 children had duplicate records in all 4 studies.

After removing 350 MTX and 605 TNFi duplicate entries, the final datasets contain 2899 and 2401 unique MTX and TNFi patients respectively; 1018 are in both datasets having received both treatments. Missingness across core outcome variables ranged from 10% (active joint count MTX timepoint 2) to 60% (physician VAS TNFi timepoint 2) and was not improved through combining datasets with duplicate entries. Specificity in some variables was lost to allow integration by combining data using least common denominators (e.g. ethnicity captured as Caucasian/Non-Caucasian, despite more specific categories available in some studies).

**Key learning points/Conclusion:**

Combining data across studies has achieved dataset sizes rarely seen in JIA, which is invaluable to progressing research into personalised treatments and disease outcomes.  However, losing specificity in some variables and missingness (a known challenge in observational data) and their impact on future analyses requires further consideration. Ongoing work includes identifying patients with both clinical and biological data that can be combined for more in-depth analyses. Both datasets are available for researchers to use via the CLUSTER Consortium Data Management Committee.

